# Evaluation of the bag-mediated filtration system as a novel tool for poliovirus environmental surveillance: Results from a comparative field study in Pakistan

**DOI:** 10.1371/journal.pone.0200551

**Published:** 2018-07-16

**Authors:** Nicolette Angela Zhou, Christine Susan Fagnant-Sperati, Jeffry Hiroshi Shirai, Salmaan Sharif, Sohail Zahoor Zaidi, Lubna Rehman, Jaffer Hussain, Rahim Agha, Shahzad Shaukat, Masroor Alam, Adnan Khurshid, Ghulam Mujtaba, Muhammed Salman, Rana Muhammed Safdar, Abdirahman Mahamud, Jamal Ahmed, Sadaf Khan, Alexandra Lynn Kossik, Nicola Koren Beck, Graciela Matrajt, Humayun Asghar, Ananda Sankar Bandyopadhyay, David Scott Boyle, John Scott Meschke

**Affiliations:** 1 Department of Environmental and Occupational Health Sciences, School of Public Health, University of Washington, Seattle, WA, United States of America; 2 National Institute of Health, Islamabad, Pakistan; 3 World Health Organization, Islamabad, Pakistan; 4 National Emergency Operations Center, Islamabad, Pakistan; 5 PATH, Seattle, WA, United States of America; 6 Bill & Melinda Gates Foundation, Seattle, WA, United States of America; University of Liverpool, UNITED KINGDOM

## Abstract

Poliovirus (PV) environmental surveillance (ES) plays an important role in the global eradication program and is crucial for monitoring silent PV circulation especially as clinical cases decrease. This study compared ES results using the novel bag-mediated filtration system (BMFS) with the current two-phase separation method. From February to November 2016, BMFS and two-phase samples were collected concurrently from twelve sites in Pakistan (*n* = 117). Detection was higher in BMFS than two-phase samples for each Sabin-like (SL) PV serotype (*p*<0.001) and wild PV type 1 (WPV1) (*p* = 0.065). Seventeen sampling events were positive for WPV1, with eight discordant in favor of BMFS and two in favor of two-phase. A vaccine-derived PV type 2 was detected in one BMFS sample but not the matched two-phase. After the removal of SL PV type 2 (SL2) from the oral polio vaccine in April 2016, BMFS samples detected SL2 more frequently than two-phase (*p* = 0.016), with the last detection by either method occurring June 12, 2016. More frequent PV detection in BMFS compared to two-phase samples is likely due to the greater effective volume assayed (1620 mL vs. 150 mL). This study demonstrated that the BMFS achieves enhanced ES for all PV serotypes in an endemic country.

## Introduction

Poliovirus (PV) environmental surveillance (ES) is essential to the Global Polio Eradication Initiative’s (GPEI) strategic plan [1,2]. It supplements acute flaccid paralysis (AFP) surveillance, playing an important role in the global eradication program [2–5]. As cases of wild poliovirus (WPV)-related paralysis decrease with successful vaccination campaigns, ES is crucial for monitoring low-level, silent circulation of PV [2,4,6]. ES can be used to monitor PV circulation in locations with absent or unreliable AFP surveillance [1,2], and to document PV importation [2,7,8]. Moreover, PV vaccine strains are detected frequently in environmental waters as they are shed in feces after administration of the live attenuated oral polio vaccine (OPV). For example, in a study examining the effect of vaccination schedules on viral excretion, viral shedding rates for PV types 1, 2, and 3 one week after the first OPV dose were 42%, 88%, and 58%, respectively, while one week after the third OPV dose they were 7%, 10%, and 16%, respectively [9]. This makes ES central for monitoring a decline in, or disappearance of, the vaccine Sabin-like (SL) PV strains, which is particularly relevant to ongoing polio endgame vaccine policy changes. These changes include the April 2016 switch from the trivalent OPV (tOPV) to the bivalent OPV (bOPV) through the withdrawal of Sabin PV type 2 from routine immunizations, and the proposed shift to a vaccination schedule using only the inactivated polio vaccine in the next 3–5 years [3,4,10,11].

The incidence of WPV transmission has decreased considerably over the past 20 years, with endemic WPV transmission restricted to three countries (Afghanistan, Pakistan, and Nigeria) and one serotype (type 1, WPV1) in 2018 [12,13]. To combat polio in Pakistan, the Pakistan Polio Eradication Initiative launched supplementary immunization activities (SIAs) in 1994, AFP surveillance in 1995, and ES in 2009 [14]. The ES method currently recommended by the World Health Organization (WHO) involves the collection of a 1-L grab sample and processing of a 500-mL aliquot by polyethylene glycol (PEG)/dextran two-phase separation (hereafter, two-phase method) [1,15]. Currently, ES in Pakistan uses the two-phase method, with samples collected monthly from 75 sites [16]. WPV1 was detected in 110 samples in 2017 compared to 62 in 2016, 84 in 2015, and 127 in 2014 [13]. Also, there were 8 confirmed AFP cases from WPV1 in 2017, compared to 20 in 2016, 54 in 2015, and 306 in 2014 [13]. As the number of AFP cases from PV decreases, the need for ES increases to assist with monitoring for potential silent PV circulation within the population.

To assist in polio eradication efforts by enhancing surveillance sensitivity, an in-field filtration ES method called the bag-mediated filtration system (BMFS) was developed [17]. The BMFS was designed to enable sampling and field processing of large water volumes without the use of a power source, thus eliminating the need to transport water samples to a processing laboratory. The BMFS utilizes gravity filtration for in-field primary concentration of large sample volumes (2.9 to >10 L, depending on the water source) [17]. As this method allows for processing of 6 to >20 times more volume than the two-phase method, the BMFS results in an increased effective volume assayed after processing [18], and increased sensitivity for poliovirus detection. In addition, on-site filtration reduces transport logistics, as only the small cartridge filter requires cold-chain shipment to the processing laboratory, rather than a large volume of biohazardous water requiring cold chain. The next step in the BMFS development involved field testing to examine its use as an environmental sampling technology.

This study monitored WPV, PV vaccine strains, and vaccine-derived PV (VDPV) in environmental samples from the polio endemic country of Pakistan. The objective of this study was to compare BMFS ES results with those from sequentially collected, co-located two-phase samples processed by the WHO method [1,15]. This enabled a direct comparison between these two sampling and concentration methods and facilitated monitoring of SL PV type 2 (SL2) disappearance from the environment after the switch from the tOPV to the bOPV. Additionally, this study allowed the global eradication program to respond to BMFS research findings in real-time with OPV SIAs.

## Materials and methods

### Study plan

**Fig 1:**
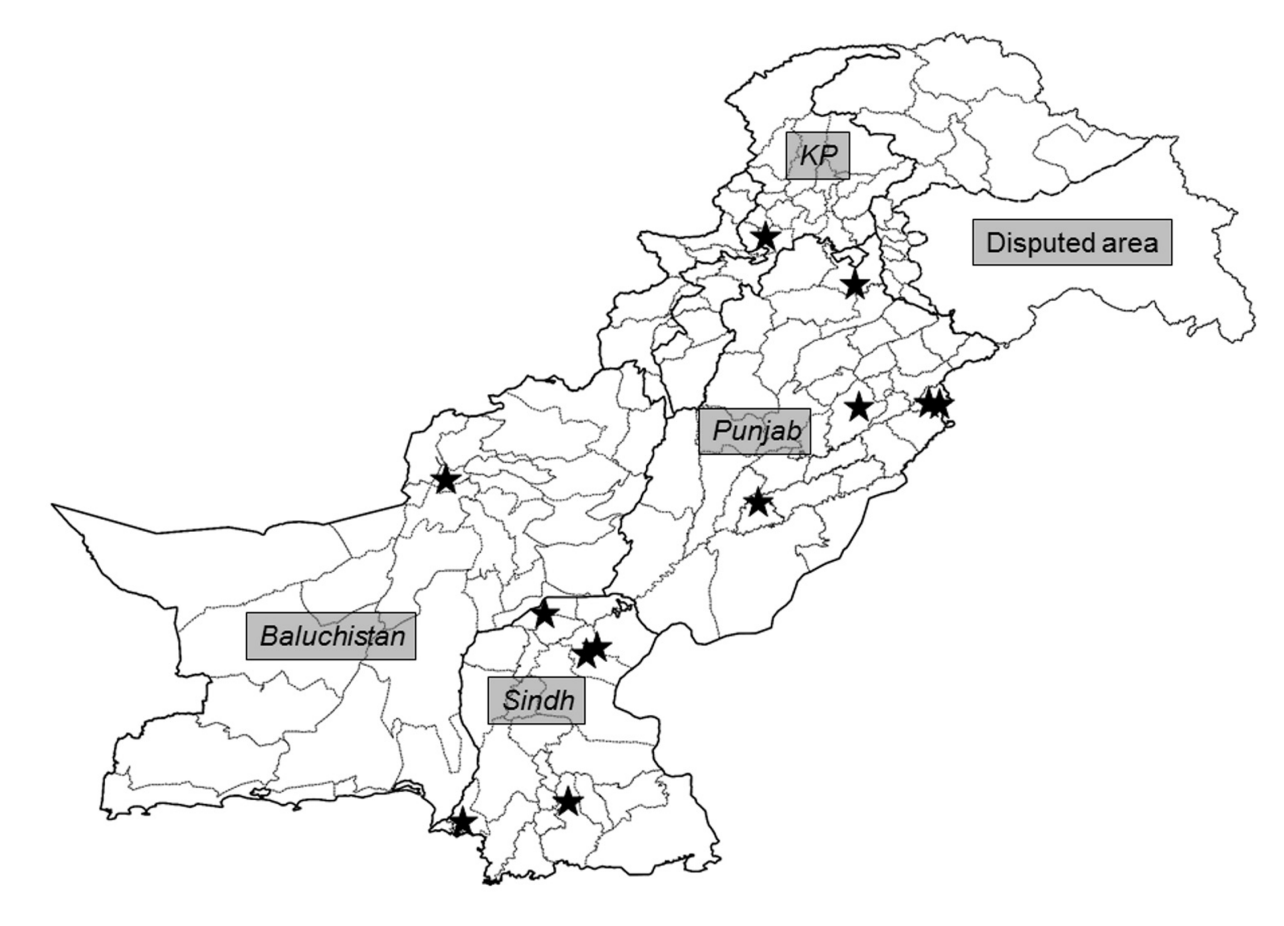
Location of 12 study sites in 10 Pakistani districts. KP is Khyber Pakhtunkhwa province.

From February to November 2016, BMFS and two-phase samples were sequentially collected once per month from 12 study sites and analyzed for PV (*n* = 117). Two-phase samples were collected first, and BMFS samples were collected within 5 minutes in the same location (1-meter radius). Samples were obtained from pumping station inlets (8 sites) and open drainage systems (4 sites) located in 10 districts within four Pakistani provinces (Table 1 and Fig 1). A memorandum of understanding was signed between the National Institute of Health (NIH) in Islamabad, National Emergency Operations Center (NEOC), and PATH for the operation of this study. Samples were collected from sites designated by the GPEI on public, not-protected land. No endangered or protected species were sampled. The total population contributing to wastewater at a given site ranged from <150,000 to >1,300,000 people, and the population under the age of five ranged from <20,000 to ~200,000 children. Sample collection began between 07:00–10:55, with an average start time of 08:37±00:08 (*n* = 116; Table 1 and S1 Fig).

**Table 1:** Location and type of sampling sites in Pakistan.

District	Collection site (geographic coordinates)	Type of sampling site	Samples collected	Average collection start time (hh:mm)^a^	Average volume filtered (L)^a^	Average time filtered (min)^a^	Median time filtered (min)
*Sindh*							
Karachi	Rashid Minhas (24.92880, 67.08753)	Open drainage	10	09:26 ± 00:18 (*n* = 10)	5.6 ± 0.4 (*n* = 10)	38 ± 14 (*n* = 7)^b^	35 (*n* = 7)^b^
Sukkur	Makka pumping station (27.71955, 68.81827)	Pumping station	9	07:42 ± 00:15 (*n* = 9)	5.0 ± 0.0 (*n* = 8)^b^	68 ± 15 (*n* = 7)^b^	60 (*n* = 7)^b^
Sukkur	Miani pumping station (27.68977, 68.86129)	Pumping station	9	07:40 ± 00:16 (*n* = 9)	5.0 ± 0.0 (*n* = 9)	64 ± 13 (*n* = 8)^b^	58 (*n* = 8)^b^
Hyderabad	Tulsidas pumping station (25.38018, 68.37504)	Pumping station	10	09:41 ± 00:24 (*n* = 10)	5.5 ± 0.3 (*n* = 10)	44 ± 10 (*n* = 10)	43 (*n* = 10)
Jacobabad	Sadar pumping station (28.16578, 68.25573)	Pumping station	10	08:10 ± 00:10 (*n* = 10)	5.1 ± 0.7 (*n* = 10)	150 ± 38 (*n* = 8)^b^	135 (*n* = 8)^b^
*Punjab*							
Lahore	Main outfall well-1 (31.57263, 74.29150)	Pumping station	10	08:30 ± 00:15 (*n* = 10)	5.8 ± 0.3 (*n* = 10)	51 ± 11 (*n* = 9)^b^	55 (*n* = 9)^b^
Lahore	Main outfall well-2 (31.57263, 74.29150)	Pumping station	10	08:57 ± 00:17 (*n* = 10)	5.9 ± 0.2 (*n* = 10)	47 ± 17 (*n* = 9)^b^	45 (*n* = 9)^b^
Rawalpindi	Safdarabad (33.37140, 73.02560)	Open drainage	10	08:50 ± 00:09 (*n* = 10)	5.9 ± 0.1 (*n* = 10)	186 ± 38 (*n* = 9)^b^	180 (*n* = 9)^b^
Multan	Ali town (30.16740, 71.50650)	Pumping station	10	08:33 ± 00:04 (*n* = 10)	6.2 ± 0.3 (*n* = 10)	84 ± 14 (*n* = 7)^b^	90 (*n* = 7)^b^
Faisalabad	Pump station (3) (31.45069, 73.02023)	Pumping station	10	08:36 ± 00:15 (*n* = 10)	5.9 ± 0.1 (*n* = 10)	154 ± 36 (*n* = 8)^b^	128 (*n* = 8)^b^
*Khyber Pakhtunkhwa*							
Peshawar	Shaheen Muslim town (34.00763, 71.61781)	Open drainage	9	09:51 ± 00:30 (*n* = 8)^b^	4.5 ± 0.5 (*n* = 9)	89 ± 20 (*n* = 9)	75 (*n* = 9)
*Baluchistan*							
Quetta	Jamia Salfia (30.25087, 66.96769)	Open drainage	10	07:29 ± 00:18 (*n* = 10)	4.2 ± 0.8 (*n* = 10)	121 ± 32 (*n* = 8)^b^	120 (*n* = 8)^b^

^a^ ± represents 95% confidence intervals on the mean.

^b^ data not recorded for some sampling events

### BMFS samples

**Fig 2:**
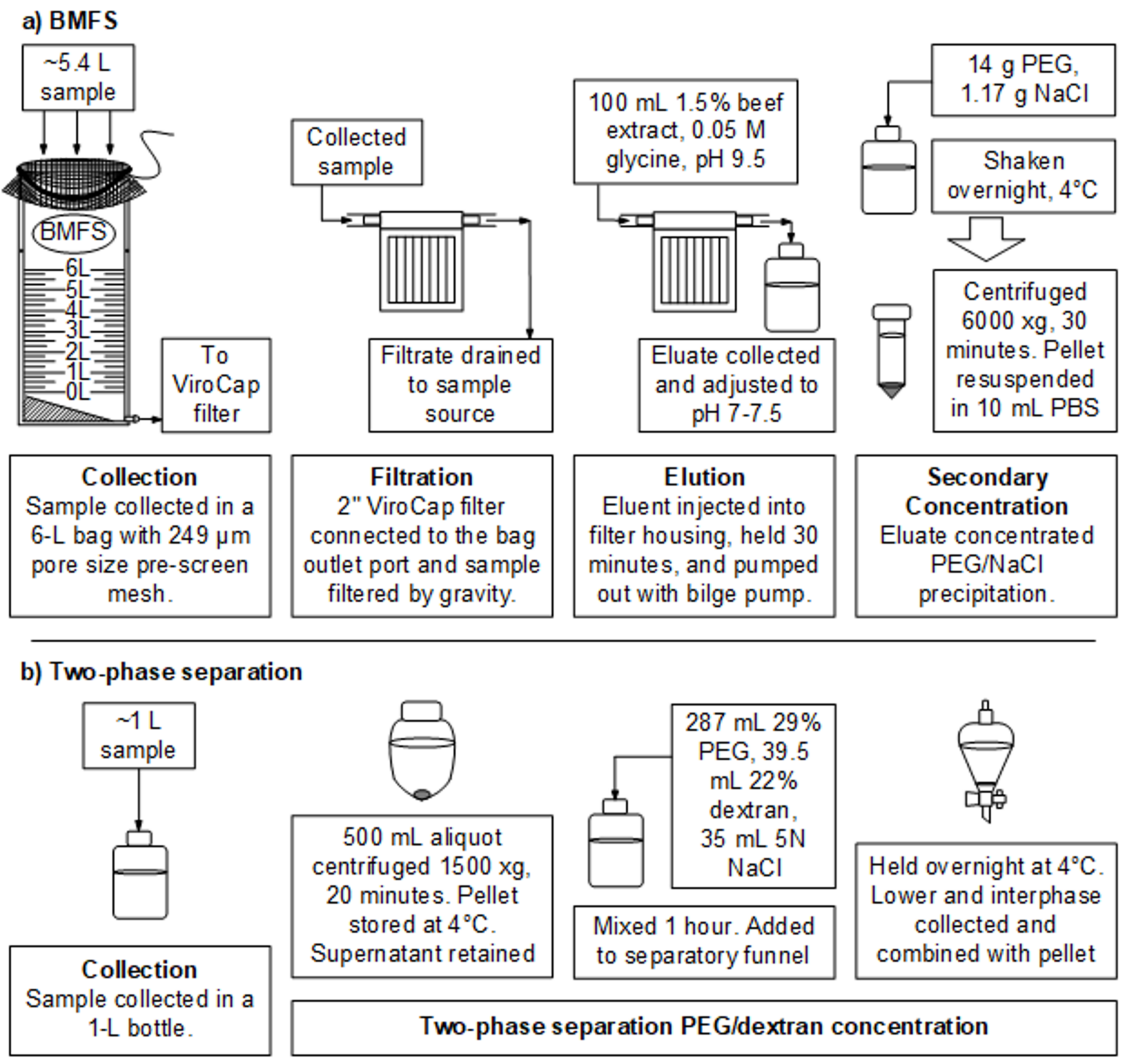
**Workflow of the a) bag-mediated filtration system (BMFS) and b) two-phase method.** PEG is polyethylene glycol. NaCl is sodium chloride. PBS is phosphate-buffered saline.

BMFS samples were collected and processed as described previously (Fig 2A) [18]. Briefly, samples were collected in a 6-L sampling bag with a pre-screen mesh (249-μm pore size) over the opening. Samples were filtered onsite at 11 sites. However, due to security concerns at the Quetta site, a “bucket” protocol was used to transport samples to a secure location prior to filtration. An insulated bucket was prepared before sample collection by placing two pre-frozen cold packs into a bucket, securing the water-tight lid, and placing the bucket in a Kool Bucket^TM^ (Kool Buckets LLC, Huntington Station, NY, USA) insulated cover. The sample was then collected, and the sampling bag was secured tightly with a bag clip, sanitized with 70% ethanol, and placed into the insulated bucket for secondary containment during travel to a secure location. For filtration of all samples, the collection bag was hung on a custom-made tripod (BoundaryTEC, Minneapolis, MN, USA). A 2” ViroCap^TM^ filter (Scientific Methods, Inc., Granger, IN, USA) was attached to the bag’s outlet port and the sample filtered by gravity. The ViroCap filter was encased within a custom-designed, injection-molded, clear polycarbonate sump (Proto-labs, Maple Plain, MN, USA) and commercially available reinforced polypropylene lid (Pentek, Inc., Upper Saddle River, NJ, USA) [18]. The average volume filtered was 5.4±0.1 L (*n* = 116), and the average filtration time was 91±11 minutes with a median of 75 minutes (*n* = 99; S2 Fig). After filtration, the filter was transported under cold chain in a routine stool carrier to the NIH in Islamabad and received within 48 hours. To improve virus survival during storage prior to elution, filters were treated with a preservative mixture as described previously (*n* = 85/117) [19]. Briefly, a 100-mL preservative mixture (2% sodium benzoate [Alfa Aesar, Ward Hill, MA, USA] and 0.2% calcium propionate [TCI America, Portland, OR, USA]) was injected into the filter inlet, held for 20 minutes, and then pumped through the filter outlet using a peristaltic pump (Cole Parmer, Vernon Hills, IL, USA). ViroCap filters were eluted with 100 mL 1.5% beef extract (BBL^TM^ Beef Extract powder; Becton, Dickinson and Company, Sparks, MD, USA) and 0.05 M glycine (Merck, Darmstadt, Germany) solution at pH 9.5 [18]. The eluent was injected into the filter inlet and incubated for 30 minutes before recovering the eluate through the filter outlet using a peristaltic pump. The recovered eluate was pH-adjusted to 7.0–7.5 and further concentrated using the PEG 8000/NaCl precipitation secondary concentration method [18]. Briefly, PEG 8000 (14 g; AMRESCO, Solon, OH, USA) and NaCl (1.17 g; Merck) were added to the eluate and dissolved by vigorous shaking. Samples were shaken (200 rpm, 4°C, overnight), centrifuged (6000 x *g*, 4°C, 30 minutes), and the pellet was resuspended in a final volume of 10 mL phosphate-buffered saline, pH 7.4.

BMFS samples were screened for WPV1, WPV type 3 (WPV3), SL PV type 1 (SL1), SL2, SL PV type 3 (SL3), VDPV, and non-polio enterovirus (NPEV). Samples were analyzed using the WHO algorithm by virus isolation using L20B and Human Rhabdomyosarcoma (RD) cells followed by intratypic differentiation (ITD) using real-time reverse transcription (RT)-polymerase chain reaction (PCR) [1]. Real-time RT-PCR was performed using the Poliovirus ITD 4.0/4.1 rRT-PCR Kit (Centers for Disease Control and Prevention, Atlanta, GA, USA) using an Applied Biosystems® 7500 thermocycler (Applied Biosystems, Foster City, CA, USA). Viral protein (VP) type 1 gene sequencing was performed on wild type and PV type 2 isolates using capillary electrophoresis-based Sanger sequencing and phylogenetic analysis was performed using MATLAB® (The MathWorks, Inc., Natick, MA, USA) [20].

### Two-phase samples

Two-phase samples were collected according to WHO protocols (Fig 2B) [1].One liter grab samples were collected and transported under cold chain to the NIH in Islamabad and received within 48 hours. A 500-mL aliquot of each sample was concentrated using the two-phase PEG/dextran aqueous polymer separation method as described previously [1]. Briefly, samples were centrifuged to pellet debris, then PEG 6000, dextran, and NaCl were added to the supernatant, mixed for one hour, added to a separation funnel, and held overnight at 4°C. The lower phase and interphase in the separation funnel were collected, combined with the pellet, and chloroform extracted. The upper aqueous phase was collected (10 mL) and samples were analyzed using the WHO algorithm [1]. Two-phase samples were not further processed by a secondary concentration method.

### Statistical analyses

The means and 95% confidence intervals (CIs) were calculated for the sample volume and filtration time. For matched BMFS and two-phase samples, the McNemar mid-*p* test was used to determine the significance of the difference [21,22], and results were considered significant with a mid-*p*-value <0.05. The odds ratio (OR) was calculated to determine the odds that PV was detected more frequently by the BMFS than by the two-phase method, and 95% CIs were calculated for the OR. For samples collected before and after the OPV switch, the Pearson’s chi-squared test was used to determine the likelihood that the differences seen in PV detection were due to chance. The OR was calculated to determine the odds that SL2 was detected more frequently during tOPV use than during bOPV use. Calculations are shown in the Supplementary Information.

## Results

### WPV1 detection

**Fig 3:**
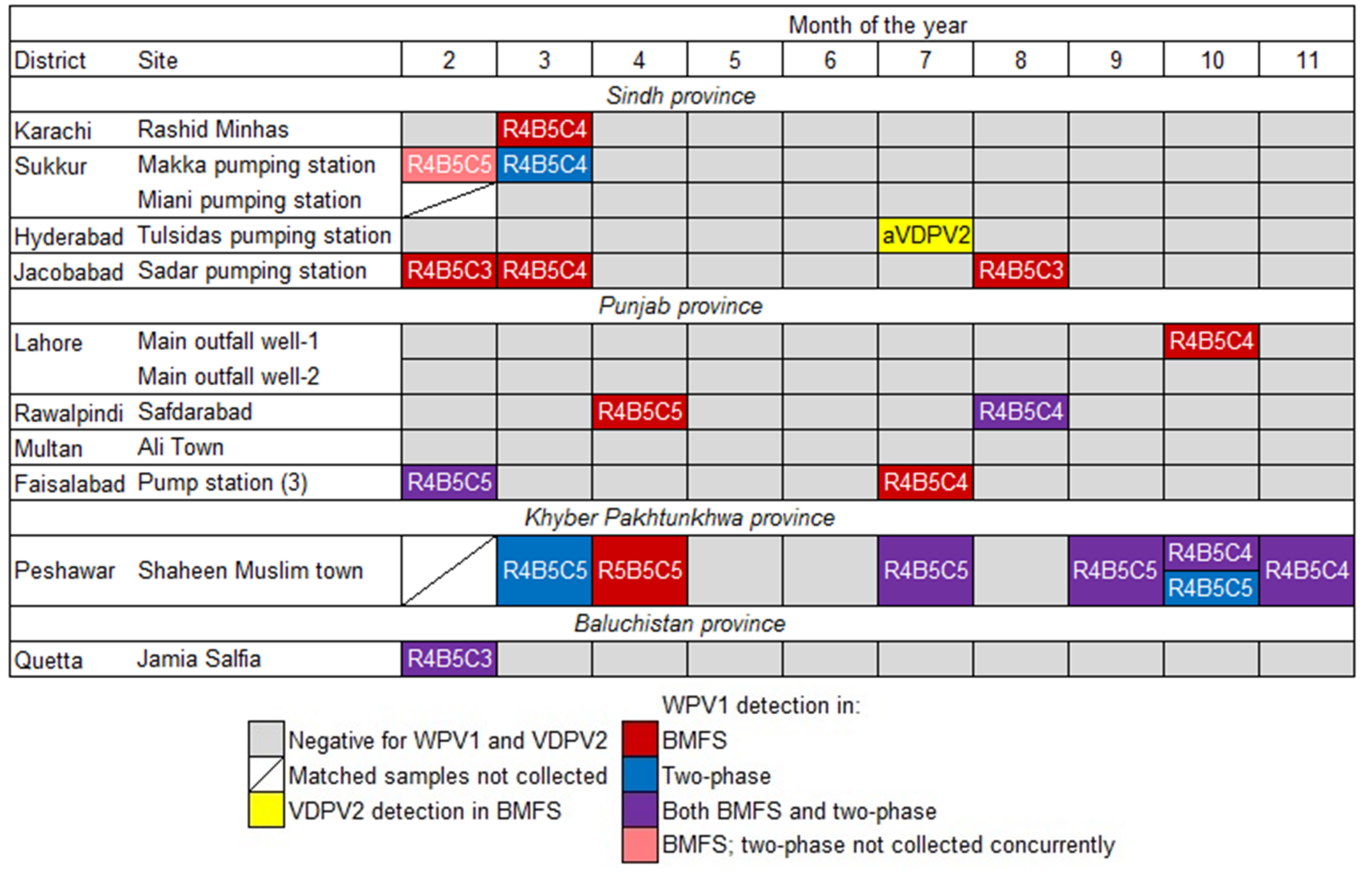
Detection of wild poliovirus type 1 (WPV1) and vaccine-derived poliovirus type 2 (VDPV2) in bag-mediated filtration system (BMFS) and two-phase samples from 12 study sites in 10 Pakistani districts from February-November 2016. The genetic cluster of WPV1 isolates is shown. The VDPV2 detected was an ambiguous VDPV2 (aVDPV2) with 12 nucleotide changes.

For matched BMFS and two-phase samples (*n* = 117), WPV1 was detected in 15 BMFS (12.8%) and 9 two-phase samples (7.7%) (Table 2). Eight of these samples were discordant in favor of the BMFS, while two were discordant in favor of the two-phase method (Fig 3). Of the eight sampling events in which the BMFS detected WPV1 and the two-phase method did not, five occurred in three districts at sites where the two-phase method did not detect WPV1 at any time-point during this study. The other three of these sampling events occurred in three districts at sites where the two-phase method detected WPV1 at a different time-point during this study. For the two sampling events in which the two-phase method detected WPV1 and the BMFS did not, they occurred in two districts at sites where the BMFS detected WPV1 at a different time-point during this study. WPV1 was detected in the February BMFS sample from the Sukkur–Makka pumping station site, although the BMFS and two-phase samples were not collected concurrently due to logistical supply issues. Therefore this data point was excluded from statistical analyses.

**Table 2:** PV detection in matching BMFS and two-phase samples as measured by ITD after virus amplification on L20B and RD cells.

	WPV1 (%)	SL1 (%)	SL2 (%)	SL3 (%)	*n*
BMFS	12.8	60.7	25.6	74.4	117
Two-phase	7.7	33.3	16.2	50.4	117
Discordant	8.5	37.6	16.2	37.6	117
*BMFS*	*6*.*8*	*32*.*5*	*12*.*8*	*30*.*8*	*117*
*Two-phase*	*1*.*7*	*5*.*1*	*3*.*4*	*6*.*8*	*117*

PV, poliovirus; BMFS, bag-mediated filtration system; ITD, intratypic differentiation; RD, Human Rhabdomyosarcoma; WPV1, wild poliovirus type 1; SL1, Sabin-like poliovirus type 1; SL2, Sabin-like poliovirus type 2; SL3, Sabin-like poliovirus type 3; Discordant indicates matching BMFS and two-phase samples resulted in discordant results, and is divided into sample subsets: positive by the BMFS and positive by the two-phase method

Three different WPV1 genetic clusters were detected throughout this study: R4B5C3, R4B5C4, and R4B5C5 (Fig 3). At all sites where WPV1 was detected more than once during this study, more than one WPV1 cluster was isolated from the same site in five different districts, *i*.*e*., clusters R4B5C4 and R4B5C5 were detected in Sukkur–Makka pumping station, Rawalpindi, Faisalabad, and Peshawar and clusters R4B5C3 and R4B5C4 were detected in Jacobabad. From the Sukkur–Makka pumping station site, R4B5C5 was detected in the February BMFS sample (no matching two-phase sample) and R4B5C4 was detected in the March two-phase sample. In Rawalpindi, both BMFS and two-phase samples detected R4B5C4 during the August sampling event, while only the BMFS detected R4B5C5 during the April sampling event. Also, in Faisalabad, both BMFS and two-phase samples detected R4B5C5 during the February sampling event, while only the BMFS detected R4B5C4 during the July sampling event. In general, when both BMFS and two-phase samples detected WPV1, they were from the same genetic cluster. However, in Peshawar, the October two-phase sample detected both R4B5C4 and R4B5C5, while the BMFS sample only detected R4B5C4.

The WPV1 detection frequency varied by location (Fig 3). WPV1 was most frequently detected in Peshawar, with a positive detection in 67% of sampling days (*n* = 9) by BMFS and/or two-phase methods. In contrast, WPV1 was detected in Jacobabad during 30% of sampling days (*n* = 10), in Sukkur–Makkka pumping station, Rawalpindi, and Faisalabad during 20% of sampling days (*n* = 10 per site), and in Karachi, Lahore–Main outfall well-1, and Quetta during 10% of sampling days (*n* = 10 per site). No WPV1 was detected in Sukkur–Miani pumping station, Hyderabad, Lahore–Main outfall well-2 or Multan.

### Comparison of PV detection in matched BMFS and two-phase samples

**Fig 4:**
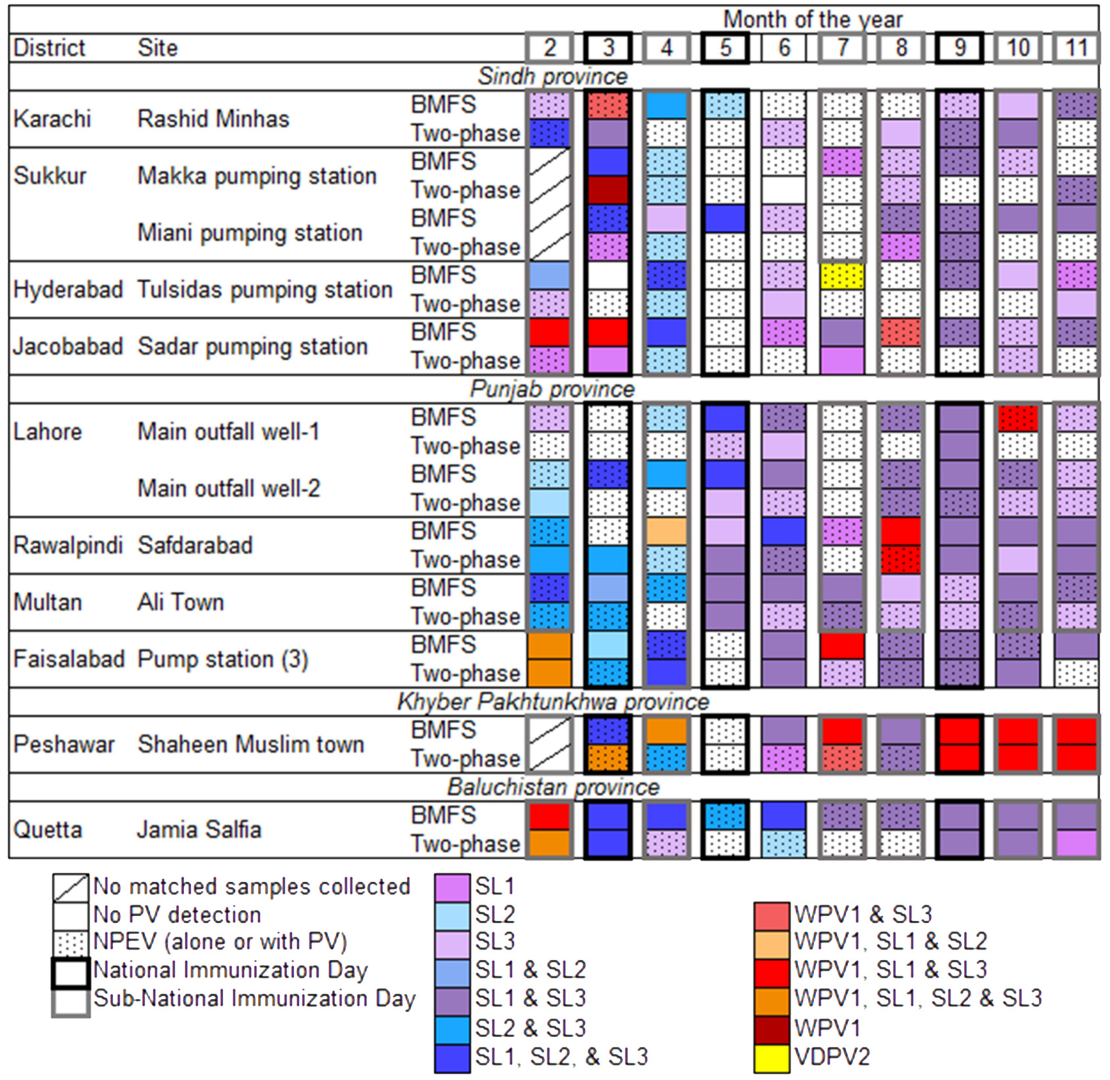
Detection of poliovirus (PV) in bag-mediated filtration system (BMFS) and two-phase samples from 12 study sites in 10 Pakistani districts from February-November 2016. NPEV is non-polio enterovirus. SL1 is Sabin-like poliovirus type 1. SL2 is Sabin-like poliovirus type 2. SL3 is Sabin-like poliovirus type 3. WPV1 is wild poliovirus type 1. VDPV2 is vaccine-derived poliovirus type 2.

PV was detected in a majority of matched BMFS and two-phase samples (*n* = 117), with WPV1, SL1, SL2, SL3, or VDPV type 2 (VDPV2) detected in 85.5% of BMFS and 63.2% of two-phase samples (Fig 4). WPV3 was not detected throughout the study. Prior to the switch from tOPV to bOPV, SL2 and SL3 were the most frequently detected PV in BMFS samples (69.7% each, *n* = 33), while SL2 was the most frequently detected in two-phase samples (54.5%, *n* = 33). After the switch to bOPV however (*n* = 84), SL3 was the most frequently detected PV in both BMFS and two-phase samples, followed by SL1, WPV1, then SL2. Often, multiple PVs were detected in a single sample, with mixtures detected in 63.2% of BMFS and 33.3% of two-phase samples (Fig 4).

PV was detected more frequently in BMFS samples than in two-phase samples for WPV1 and all SL serotypes (Table 2). Discordance between matched samples typically favored the BMFS, and the odds favored PV detection in BMFS samples over two-phase samples for WPV1 and all SL serotypes (Table 3). An ambiguous VDPV2 with 12 nucleotide changes in the VP1 region was also detected in one BMFS sample during this study (Fig 3) [23]. No VDPV2 was detected by the two-phase method during this study.

**Table 3:** Comparison of PV detection in matching BMFS and two-phase samples as measured by ITD after virus amplification on L20B and RD cells.

	Odds ratio^a^	95% confidence interval	*p*-value^b^
WPV1	4.0	0.79, 19	0.065
SL1	6.3	2.7, 15	5.4x10^-7^
SL2	3.8	1.2, 11	0.001
SL3	4.5	2.1, 9.7	1.5x10^-5^

PV, poliovirus; BMFS, bag-mediated filtration system; ITD, intratypic differentiation; RD, Human Rhabdomyosarcoma; WPV1, wild poliovirus type 1; SL1, Sabin-like poliovirus type 1; SL2, Sabin-like poliovirus type 2; SL3, Sabin-like poliovirus type 3

^a^ calculated by number of samples discordant in favor of the BMFS divided by the number of samples discordant in favor of the two-phase method

^b^ calculated by the McNemar mid-*p* test

### SL2 detection

The switch from tOPV to bOPV provided further opportunity to assess the performance of the BMFS and investigate SL2 persistence in the population. After the switch, there was less frequent detection of SL2 in both BMFS (*p*<0.0001, Pearson’s χ^2^ test) and two-phase samples (*p*<0.0001). No statistical difference in SL1, SL3, or WPV1 detection was observed after the switch in either BMFS (*p* = 0.09, 0.39, and 0.47, respectively) or two-phase samples (*p* = 0.26, 0.66, and 0.50, respectively). Before the switch in April 2016, SL2 was detected in a majority of BMFS and two-phase samples (Table 4). After the switch, SL2 was detected during 7 of 84 sampling events. Of these 7 sampling events, SL2 was detected in 1 two-phase and all 7 BMFS samples, resulting in more frequent SL2 detection in BMFS than two-phase samples after the switch (*p* = 0.016, McNemar mid-*p* test; Table 4). At 6 of the 12 sites, the final SL2 detections occurred in April, immediately before the switch to bOPV. For the remaining sites, the final SL2 detections occurred in May (Karachi, Sukkur–Miani pumping station, and both Lahore sites) and June (Rawalpindi and Quetta). The final SL2 detection of the study occurred on June 12, 2016 in both BMFS and two-phase samples from Quetta.

**Table 4:** SL2 detection in BMFS and two-phase samples as measured by ITD after virus amplification on L20B and RD cells.

Sample	SL2 (%)	*n*
*tOPV (February 2016 –April 2016)*		
BMFS	69.7	33
Two-phase	54.5	33
*bOPV (May 2016 –November 2016)*		
BMFS	8.3	84
Two-phase	1.2	84

SL2, Sabin-like poliovirus type 2; BMFS, bag-mediated filtration system; ITD, intratypic differentiation; RD, Human Rhabdomyosarcoma; tOPV, trivalent oral polio vaccine; bOPV, bivalent oral polio vaccine

## Discussion

As Pakistan is one of the three polio-endemic countries in the world, more accurate surveillance of WPV1 circulation in this area is critical to achieve and confirm global eradication. Although multiple WPV1 clusters and lineages still circulate and paralyze children, the overall case rate is diminishing in Pakistan and Afghanistan [12]. The number of detected genetic clusters from environmental samples or clinical cases also decreased from 21 clusters in 2014 to 12 in 2015 and 8 in 2016. Currently, there are three primary WPV1 reservoirs that comprise this area: Karachi, Quetta-Greater Kandahar (including Pakistan’s Pishin and Killa Abdulla districts and Afghanistan’s Kandahar and Helmand provinces), and Peshawar-Khyber-Nangarhar (including Pakistan’s Peshawar district and Khyber Agency and Afghanistan’s Nangarhar province). A primary challenge for the GPEI within this region is the large population that travels frequently between and within Pakistan and Afghanistan [24], increasing the likelihood of importing PV from reservoir districts to other areas and introducing WPV1 to new susceptible populations [24,25].

This study enabled the GPEI to report upon and respond to WPV1 detections in BMFS samples, and also to further understand the presence and extent of WPV1 circulation in Pakistan. WPV1 was detected in 16 BMFS samples during this study and these results were reported, along with 9 positive two-phase results, by the GPEI. Additionally, due to the detection of WPV1 in Faisalabad in the February BMFS and two-phase samples and in the July BMFS sample, bOPV SIAs were conducted in Faisalabad in March and August 2016, respectively. Of the 16 BMFS samples positive for WPV1, 7 WPV1 isolates were collected from reservoir areas with evidence of indigenous circulation (Karachi, Peshawar, and Quetta; Fig 3), and the remaining 9 were indirectly or directly linked to these high-risk core reservoir areas based on phylogenetic analysis with a homology greater than 95% (S1 Table). Of the 9 two-phase samples positive for WPV1, 6 were collected from reservoir areas with indigenous circulation, and the remaining 3 linked to these high-risk areas (S1 Table). For example, Faisalabad isolates from the February BMFS and two-phase samples were directly linked to a Nangarhar, Afghanistan AFP WPV1 case (cluster R4B5C5; homology >95%). Similarly, the Jacobabad isolate from the March BMFS sample directly corresponded to a WPV1 isolated from Shikarpur AFP cases, ultimately linking to a virus circulating in the Quetta-Greater Kandahar region (cluster R4B5C4; homology >95%). Direct viral importation by human migration from a reservoir to a non-endemic community was demonstrated by the Faisalabad isolate from the July BMFS sample, which was linked to a Karachi indigenous virus within cluster R4B5C4. Similarly, the Jacobabad isolate from the February BMFS sample was directly related to a Quetta-Greater Kandahar virus within cluster R4B5C3. Finally, Rawalpindi isolates from the August BMFS and two-phase samples were within cluster R4B5C4 and homologous with Karachi environmental samples, and the WPV1 isolated from the March Sukkur–Makka pumping station two-phase sample directly was related to a Karachi virus (cluster R4B5C4; >95% homology).

This study highlighted enhanced PV detection when using the BMFS in an endemic country. PV was detected more frequently in BMFS than two-phase samples (Table 2), likely due to the greater volumes filtered with the BMFS (5.4±0.1 L) than processed by the two-phase method (500 mL). After processing, BMFS samples are concentrated ~540x compared to 50x for two-phase samples, resulting in an increased sensitivity for poliovirus detection. As 3 mL are inoculated onto tissue culture, this results in a greater effective volume assayed of the original sample (1620 mL BMFS vs. 150 mL two-phase). Mixtures of PV serotypes were also more frequently detected in BMFS than two-phase samples (Fig 4), underlining the ability of the BMFS to isolate multiple PV serotypes present at disparate levels in the environment. Finally, the more frequent WPV1 detection in BMFS samples suggests that sites where only two-phase samples are collected may not be as successful in detecting circulation as sites where BMFS samples are collected.

To eliminate the risk of circulating VDPV and vaccine-associated paralytic poliomyelitis caused by SL2, the tOPV to bOPV (types 1 and 3) switch was implemented globally between April 17 and May 1, 2016 [26,27]. In Pakistan, SL2 was withdrawn from routine use on April 25 [12]. During the period of this study, tOPV was used at all sites during Pakistan’s National Immunization Day from March 14–17, 2016, and bOPV during the remainder of vaccine campaigns. The length of this study spanned the time prior to, during, and after this unprecedented global switch, enabling a direct comparison of SL2 detection frequency throughout this unique period of vaccine policy transition. The less frequent and declining trend of SL2 detection in BMFS and two-phase samples collected from May to November 2016, compared to samples collected from February to April, demonstrated the completeness of type 2 OPV withdrawal.

This study demonstrated the feasibility of using the BMFS for PV ES with sustainable BMFS sampling and processing timeframes. The median time between collection and completion of secondary concentration was 6.0±0.7 days (95% CI; *n* = 111), with a range of 1–19 days and 84% of samples processed within 7 days. This is within the current GPLN standard for PV environmental surveillance samples, which recommends that samples are concentrated within 7 days to allow for a total of 21 days between collection to completion of virus isolation [1]. The well-established courier service for PV ES sample transport to the NIH in Islamabad contributed to rapid processing, with 97 of 98 BMFS samples shipped the day of collection and an average shipping time of 1.8±0.3 days (95% CI; *n* = 98). This indicates that results for BMFS samples can be obtained within the same timeframe as two-phase samples, and within the timeframe recommended by the WHO.

This study and the tool evaluated have limitations, including the inherent virus variability in environmental samples, time spent in-field, and the complexity and cost of the current BMFS method. The study was designed to allow for direct comparison between the BMFS and two-phase methods. However, while the two sample types are collected as close together as possible, there may be differences in the viruses present between samples, and virus isolation in cell culture inherently results in variability. This may contribute to discordance in PV detection between matched BMFS and two-phase samples. The median filtration time was 75 minutes (*n* = 99), which can be problematic at security-compromised sites and/or at sites where temperatures can reach >50°C. In these situations, the “bucket” protocol and/or an additional protocol modification (filtering 3 L or for 40 minutes) should be considered as future options for ES teams using the BMFS, as they reduce in-field time and enable continued sequential collection of BMFS and two-phase samples. Issues with the BMFS can arise at sampling sites with low flows. While this was not an issue during this study, it has been observed in other locations. In these cases, sampling occurred with a bucket, and the sample was poured into the BMFS bag. This issue could be addressed by substituting the metal collar for a more flexible bag opening to increase the available surface area at the lower portion of the collection bag mouth. Additionally, the BMFS is a relatively more complicated field method than two-phase sample collection, requiring additional training of field staff. Over the study period, recall and adherence to the BMFS protocol was generally consistent. Due to field sampling components, the BMFS will be more expensive than the two-phase method, however these costs will be balanced by reduced costs and time in the laboratory. Optimization and commercialization of the BMFS kit to simplify field sampling and reduce costs is ongoing, and future work will focus on conducting a full cost comparison between the BMFS and two-phase methods. Despite potential limitations in implementing the BMFS, the benefit of increased sensitivity outweighs these concerns. This is evidenced by direct viral importation by human migration from a reservoir to a non-endemic community and by implementation of vaccination campaign responses by the global program in response to WPV1 detection using the BMFS.

In conclusion, the BMFS can be used for improved PV detection in environmental samples. This study yielded novel and important data on PV detection dynamics in an endemic setting. Additionally, new BMFS detections of WPV resulted in vaccination campaign responses by the global program, even while the BMFS was being evaluated. The study period ensured that SL2 transmission was monitored before, during, and after the unprecedented globally synchronized OPV switch. Observations about the change in SL2 detection frequency enhanced the GPEI programmatic implications of the study. This study demonstrated the feasibility of the BMFS as an effective ES tool for all types of PV. Since only the small cartridge filter requires shipment to a reference laboratory, and with the benefits of the “bucket” protocol, the BMFS is feasible in a broad range of potential situations, including conflict or high-risk zones. Moreover, in this post-switch era of the polio endgame, the BMFS would be beneficial for outbreak response monitoring following monovalent OPV type 2 use. Future work should focus on exploring new settings for BMFS use, such as other endemic or outbreak countries, given the demonstrated enhanced PV detection. Exploring integrative approaches of environmental surveillance for multiple pathogens could also be an important next step for BMFS use to strengthen polio transition planning and sustain detection capabilities for long-term.

## Supporting information

S1 Fig BMFS sample collection start time. (DOCX)

S2 Fig BMFS sample filtration time. (DOCX)

S1 Table Wild poliovirus type 1 isolates from BMFS and two-phase samples. (XLSX)

S2 Table PV detection data tables. (XLSX)

S3 Table Chain of custody form tables. (XLSX)

S1 File BMFS sample processing and statistical analysis methods. (DOCX)
