# Supplementary material for: Evaluation of the bag-mediated filtration system as a novel tool for poliovirus environmental surveillance: Results from a comparative field study in Pakistan
Source: PLoS One. 2018 Jul 16;13(7):e0200551. doi: 10.1371/journal.pone.0200551 (PMC6047795; doi:10.1371/journal.pone.0200551)
Supplement: S2 Fig — (DOCX) [file pone.0200551.s002.docx]

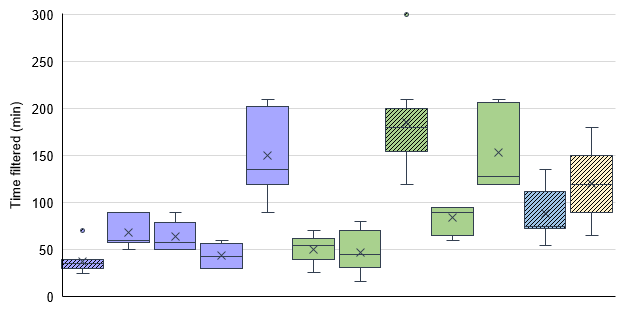


Karachi

Sukkur - Makka

pumping station

Multan

Faisalabad

Peshawar

Quetta

Lahore - Main outfall well-1

Lahore - Main outfall well-2

Rawalpindi

Jacobabad

Hyderabad

Sukkur - Miani

pumping station

*Sindh*

*Punjab*

*Khyber*

*Paktu-*

*nkhwa*

*Baluc-*

*histan*

**S2 Fig. BMFS sample filtration time.** Box and whisker plot: *upper*, *middle*, and *lower box lines* show the first, second, and third quartiles, respectively; markers ‘x’ show the mean; and error bars show the range. Hatched boxes represent open drainage sampling sites, and solid boxes represent pumping station sampling sites
