# Supplementary material for: Evaluation of the bag-mediated filtration system as a novel tool for poliovirus environmental surveillance: Results from a comparative field study in Pakistan
Source: PLoS One. 2018 Jul 16;13(7):e0200551. doi: 10.1371/journal.pone.0200551 (PMC6047795; doi:10.1371/journal.pone.0200551)
Supplement: S1 File — (DOCX) [file pone.0200551.s006.docx]

SUPPORTING INFORMATION

Evaluation of the bag-mediated filtration system as a novel tool for poliovirus environmental surveillance: Results from a comparative field study in Pakistan

Nicolette Angela Zhou^1^, Christine Susan Fagnant-Sperati^1^, Jeffry Hiroshi Shirai^1^, Salmaan Sharif^2^, Sohail Zahoor Zaidi^2^, Lubna Rehman^2^, Jaffer Hussain^2^, Rahim Agha^3,4^, Shahzad Shaukat^2^, Masroor Alam^2^, Adnan Khurshid^2^, Ghulam Mujtaba^2^, Muhammed Salman^2^, Rana Muhammed Safdar^2,4^, Abdirahman Mahamud^3,4^, Jamal Ahmed^3,4^, Sadaf Khan^5^, Alexandra Lynn Kossik^1^, Nicola Koren Beck^1^, Graciela Matrajt^1^, Humayun Asghar^3^, Ananda Sankar Bandyopadhyay^6^, David Scott Boyle^5^, John Scott Meschke^1^*

^1^ Department of Environmental and Occupational Health Sciences, School of Public Health, University of Washington; Seattle, WA, USA

^2^ National Institute of Health; Islamabad, Pakistan

^3^ World Health Organization; Islamabad, Pakistan

^4^ National Emergency Operations Center; Islamabad, Pakistan

^5^ PATH; Seattle, WA, USA

^6^ Bill & Melinda Gates Foundation; Seattle, WA, USA

* Corresponding author

Email: jmeschke@u.washington.edu (JSM)

*Submitted to PLOS One*

**Table of contents**

[**Methods** 3](#_Toc515618749)

[*Statistical analyses* 3](#_Toc515618750)

[**References** 5](#_Toc515618751)

# **Methods**

## **Statistical analyses**

The confidence intervals (CI) on sample volume and filtration time were calculated by:

$$95\% CI=\bar{x}\pm\frac{t\times\sigma}{\sqrt{n}} (1)$$

where $\bar{x}$ is the mean, t is the critical value when the degree of freedom is (n-1) and α is 0.05, σ is the standard deviation, and n is the number of samples.

The McNemar mid-*p* test was used to determine the significance of the difference between matched samples (*i.e.*, matched BMFS and two-phase samples) [1,2]:

$$\text{mid-}\text{p}\text{-value}=\text{2}\left( \sum_{x_{12}=0}^{min\left( n_{12},n_{21} \right)} \left( \begin{matrix} n \\ x_{12} \end{matrix} \right)\left( \frac{1}{2} \right)^{n} \right)-\left( \begin{matrix} n \\ n_{12} \end{matrix} \right)\left( \frac{1}{2} \right)^{n} (2)$$

where *n_12_* is the number of samples negative by test 1, but positive by test 2, *n_21_* is the number of samples negative by test 2, but positive by test 1, *n* is the sum of *n_12_* and *n_21_*, and *x_12_* is 0, 1, …, *min(n_12_,n_21_)*. Results were considered significant with a mid-*p*-value < 0.05.

For matched samples, the odds ratio (OR) is calculated as:

$$OR=\frac{n_{12}}{n_{21}} (3)$$

where *n_12_* is the number of samples negative by test 1, but positive by test 2 and *n_21_* is the number of samples negative by test 2, but positive by test 1.

The CI on the OR is then calculated as:

$$95\% CI=e^{lnOR\pm z\times\sqrt{\frac{1}{n_{12}}+\frac{1}{n_{21}}}} (4)$$

where z is 1.96 for 95% confidence.

The Pearson’s chi-squared test was used to determine the likelihood that the differences seen in PV detection during tOPV and bOPV use were due to chance.

$$\chi^{2}=\frac{{n\left( ad-bc \right)}^{2}}{(a+b)(c+d)(a+c)(b+d)} (5)$$

where *a* is the number of samples positive for PV during tOPV use, *b* is the number of samples negative for PV during tOPV use, *c* is the number of samples positive for PV during bOPV use, and *d* is the number of samples negative for PV during bOPV use.

The odds ratio was calculated to determine the odds that SL2 was detected more frequently during tOPV use than during bOPV use (OR_OPV_).

$${OR}_{OPV}=\frac{ad}{bc} (6)$$

# **References**

1. McNemar Q. Note on the Sampling Error of the Difference Between Correlated Proportions or Percentages. Psychometrika. **1947**; 12(2):153–157.

2. Fagerland MW, Lydersen S, Laake P. The McNemar test for binary matched-pairs data: mid-p and asymptotic are better than exact conditional. BMC Med Res Methodol. **2013**; 13:91.
